# Cross-national validation of the MHQoL: psychometric evaluation and open-source tools for assessing mental health quality of life

**DOI:** 10.1136/bmjopen-2025-108598

**Published:** 2026-05-13

**Authors:** Stijn B Peeters, Frederick W Thielen, Marleen De Mul, Marjo Sinokki, Beatriz Olaya, Christina Maria Van Der Feltz-Cornelis, Leona Hakkaart-Van Roijen

**Affiliations:** 1 Erasmus School of Health Policy & Management, Erasmus University Rotterdam, Rotterdam, Netherlands; 2 Department of Health Technology Assessment, Erasmus Universiteit Rotterdam Erasmus School of Health Policy and Management, Rotterdam, Netherlands; 3 Erasmus Universiteit Rotterdam Erasmus School of Health Policy and Management, Rotterdam, Netherlands; 4 Länsirannikon Työterveys, Turku, Finland; 5 Unit of Public Health, University of Turku, Turku, Finland; 6 Universitat de Barcelona, Barcelona, Spain; 7 CIBERSAM, Madrid, Spain; 8 Autonomous University of Barcelona, Barcelona, Spain; 9 Department of Health Sciences, York Biomedical Research Institute, University of York, Heslington, York, UK; 10 Hull York Medical School, University of York, York, UK

**Keywords:** MENTAL HEALTH, HEALTH SERVICES ADMINISTRATION & MANAGEMENT, Patient Reported Outcome Measures, Psychometrics, Quality of Life, STATISTICS & RESEARCH METHODS

## Abstract

**Objectives:**

To validate the cross-national psychometric properties of the Mental Health Quality of Life questionnaire (MHQoL) and to develop an open-source toolbox for its scoring, transformation and presentation.

**Design:**

Secondary analysis of data from a multicentre international randomised controlled trial (EMPOWER).

**Setting:**

Workplace settings in small-sized and medium-sized enterprises (SMEs) and public sector organisations in Finland, Spain and the UK.

**Participants:**

The sample included 564 employees: 122 from Finland, 114 from Spain and 328 from the UK. Most were white-collar workers in SMEs or public organisations, mainly in public administration, manufacturing, health/life sciences or higher education. Women were the majority (56%–91% across countries), and mean age ranged from 43 to 48 years.

**Interventions:**

No intervention was delivered for this analysis; data were drawn from baseline assessments.

**Primary and secondary outcome measures:**

Primary outcomes were internal consistency and construct validity of the MHQoL, evaluated using Cronbach’s alpha, measurement invariance testing and multilevel analyses of associations between MHQoL dimensions and its visual analogue scale (VAS). Secondary outcomes were convergent validity, assessed through correlations between MHQoL scores and other mental health and quality of life measures (EuroQol 5-Dimension 5- level questionnaire (EQ-5D-5L), Patient Health Questionnaire-9 (PHQ-9), Generalized Anxiety Disorder-7 (GAD-7), Insomnia Severity Index, Perceived Stress Scale-4 (PSS-4), Psychosocial Risk Scale, and World Health Organization Five Well-Being Index (WHO-5)).

**Results:**

The MHQoL showed good internal consistency across countries, with Cronbach’s alpha ranging from 0.741 in Finland to 0.806 in Spain (overall α=0.787). Measurement invariance across Finland, Spain and the UK supported construct validity. Multilevel regression analyses showed associations between MHQoL dimensions and the MHQoL-VAS, with strongest contributions from Self-Image, Daily Activities, Mood and Future. Convergent validity was supported by moderate to strong correlations between MHQoL, EQ-5D-5L and related mental health measures. An open-source R package and Shiny web application (‘MHQoL Toolbox’) were developed for scoring, transformation and visualisation

**Conclusions:**

The MHQoL is a reliable and valid measure of mental health-related quality of life across countries. The MHQoL toolbox supports consistent, transparent implementation, facilitating use in research, clinical practice and economic evaluations.

**Trial registration number:**

NCT04907604.

**STRENGTHS AND LIMITATIONS OF THIS STUDY**

- Inclusion of a multicountry dataset enabled cross-national comparisons and accounted for cultural differences in workplace environments.
- Reliability and validity of the Mental Health Quality of Life (MHQoL) were assessed using multiple psychometric approaches, including internal consistency, construct validity and convergent validity.
- The study combined psychometric validation with the development of an open-source toolbox to support standardised scoring, transformation and presentation of MHQoL data.
- Participants were predominantly female, highly educated and employed in public sector or white-collar roles, which may limit the generalisability of findings to other worker populations.
- The sample was unevenly distributed across countries and no country-specific EQ-5D-5L value set was available for Finland, requiring the use of a proxy.

## Introduction

 Mental health disorders are increasingly prevalent in modern society, with global figures continuing to rise. In 2019, an estimated 970 million people, equivalent to one in every eight individuals, were living with a mental disorder worldwide.^1^ This number has risen significantly in recent years. Preliminary estimates suggest a 26% increase in anxiety disorders and a 28% rise in major depressive disorders within just 1 year.^2^ The societal economic burden associated with mental illness on society is substantial, with annual costs ranging from €54 million to €280 billion per country,^3^ totalling approximately €450 billion across the European Union (EU)^4^ and US$2.5 trillion worldwide.^5^

The workplace is also significantly affected by these disorders. Each year, 27% of EU workers report work-related stress, depression or anxiety.^6^ These conditions contribute to absenteeism, which is more affected by mental health issues than physical conditions,^6^,^9^ reduced job performance (presenteeism),^7 10^ lower Quality of Life (QoL),^10^,^13^ higher medical costs^10^ and employment difficulties.^11^ The resulting economic burden is substantial, with depression and anxiety leading to 12 billion lost workdays globally each year and an estimated €900 billion loss in productivity.^11^

To improve QoL and reduce the economic burden on society, interventions are required to enhance QoL at a reasonable expense. Economic evaluations can help identify treatments that provide the best value for money. Hence, it is essential that both costs and QoL are measured accurately to ensure a valid representation. Commonly used questionnaires like the 36-item Short-Form Health Survey (SF-36)^14^ and the five-dimension EuroQol questionnaire (EQ-5D)^15^ are, however, primarily focused on physical aspects and may not sufficiently capture the domains of mental health problems in their assessments of QoL. As a result, the impact of mental health on QoL is less represented in the outcomes of these questionnaires.^16^,^18^

To overcome this, the Mental Health Quality of Life (MHQoL)^19^ questionnaire was developed, which is a preference-based instrument designed to assess QoL using dimensions that individuals with mental health challenges identify as important and value highly. These dimensions include Self-Image, Independence, Mood, Relationships, Daily Activities, Physical Health and Hope. Hence, the MHQoL can be used alongside generic measures in economic evaluation studies such as cost-effectiveness analysis.

While previous psychometric evaluations of the MHQoL have been conducted,^19 20^ they did not specifically target the working population. However, this focus is crucial given the unique characteristics of the demographic, including the dual pressures of professional and personal responsibilities, exposure to work-related stressors and the impact of mental health on productivity and absenteeism. Mental health problems among working-age individuals are a growing concern in both public health and economic terms, contributing significantly to societal costs through reduced labour participation and increased healthcare use. Valid and practical tools to measure mental health-related QoL in this group are therefore essential to inform targeted interventions, workplace policies and economic evaluations.^21^ Furthermore, no studies have compared the MHQoL and the EQ-5D-5L in conjunction with other mental health questionnaires (eg, anxiety, depression, stress, work stress and well-being questionnaires), or cultural differences between countries. Understanding these differences is important because it provides valuable insights into how each questionnaire addresses mental health issues. Moreover, there is not a standardised tool for calculating the MHQoL. Although a manual is available, calculations must be performed manually each time, making the process time-consuming and prone to inconsistencies and errors.

The objectives of this study were twofold. First, to assess the psychometric performance of the MHQoL including reliability and validity, when applied in a working population across Finland, Spain and the United Kingdom, and to compare these findings with prior evidence from the general population by Enzing *et al*.^20^ Second, to develop a standardised, open-access toolbox for scoring, transforming and reporting MHQoL outcomes. This toolbox aimed to promote reproducibility, user-friendliness and wider uptake of the MHQoL in research and health economic practice, among users and health policy makers.

## Methods

### Data sources

Data for this study were derived from the EMPOWER trial (trial number NCT04907604), which has been described in detail elsewhere.^22^ The EMPOWER trial was a multicentre cluster randomised controlled trial conducted in workplace settings, in which organisations (clusters) were recruited and employees were invited to participate on a voluntary basis. Eligible participants were employees working in participating organisations who had access to the eHealth platform and were able to complete questionnaires in the respective country language. Recruitment was conducted through participating organisations, where employees received information about the study and were invited to enrol.

For the present analysis, cross-sectional baseline data from the trial was used. Preparation of this report was guided by the Consolidated Standards of Reporting Trials (CONSORT) checklist^23^; however, as this is a secondary analysis of baseline data from a multicentre international randomised controlled trial, full details of the trial design and methodology are reported elsewhere,^22^ and several CONSORT items were therefore not applicable.

### Population

The sample included 564 employees, derived from baseline data of the EMPOWER trial and based on the availability of complete baseline questionnaire data. While the original EMPOWER study aimed to recruit 729 participants to evaluate intervention effectiveness,^22^ the present study focuses on the psychometric evaluation of the MHQoL; therefore, this sample size was considered adequate for the present analyses. Of these participants, 122 were from Finland, 114 from Spain and 328 from the UK. Female participants predominated (56.1% in Spain, 91.0% in Finland, 82.9% in the UK). The mean age was 46.5 years in Finland (range: 21–67), 47.8 years in Spain (range 21–66) and 43.1 years in the UK (range: 23–72). Most participants were white-collar workers (~90%) and employed in the public sector, reaching 100% in Finland and the UK. In Finland, all participants worked in public administration, in Spain, the majority were in manufacturing (59.6%), with others in public administration; in the UK, most were employed in health/life sciences (82.9%) and higher education. See online supplemental appendix A, table 1 for full details.

### Instruments and outcome measures

Participants completed self-report questionnaires via the EMPOWER eHealth platform, using validated Finnish, Spanish and English versions of the instruments. The EQ-5D-5L language versions were obtained from the EuroQol Group, while the MHQoL translations were provided by Erasmus University Rotterdam.

The MHQoL includes seven dimensions (i.e. self-image, independence, mood, relationships, daily activities, physical health and hope) on a four level scale (0=very poor, 3=very good least favourable condition and 3 the most favourable.^19^ The total score ranges from 0 to 21, with higher scores indicating better mental health-related QoL. It also includes a visual analogue scale (MHQoL-VAS) from 0 (worst imaginable psychological well-being) to 10 (best). Preference-based utilities are available only for the Dutch dataset and therefore not used in the statistical analyses.

The EQ-5D-5L is a standardised instrument for assessing health-related QoL, covering five dimensions: mobility, self-care, daily activities, pain/discomfort and anxiety/depression.^24^ Each is rated on five levels of severity. Results include preference-based utility scores, Level Sum Scores (LSS, range: 5=best health to 25=worst health),^25^ and a VAS (0=worst, 100=best health).^24^ Country-specific value sets were applied^26 27^; as no Finnish value set was available, Denmark’s^28^ was used as best proxy.

The PHQ-9 is a self-assessment protocol to assess severity of depression via nine items scored 0–3, yielding a total score from 0 to 27^29^. Scores indicate severity levels: minimal (1–4), mild (5–9), moderate (10–14), moderately severe (15–19) and severe (20–27).

The GAD-7 is a 7-item scale for identifying Generalised Anxiety Disorder and assessing symptom severity.^30^ Each item is rated 0–3, with total scores from 0 (no symptoms) to 21 (severe symptoms).

The Insomnia Severity Index (ISI) is a 7-item tool evaluating issues related to falling and staying asleep, satisfaction with sleep, daytime impairment and distress about sleep problems.^31^ Each item is scored 0–4, with a total score of 0–28. Higher scores indicate more severe insomnia.^32^

The PSS-4 is a 4-item instrument measuring perceived stress.^33^ Items are rated 0 (‘never’) to 4 (‘very often’), yielding a score from 0 to 16, with higher scores indicating greater stress.

The Psychosocial Risk Scale (PRS) measures work-related stress.^34^ It includes 16 items scored from 0 (‘not at all’) to 3 (‘very stressed’), resulting in a score from 0 (no stress) to 48 (extreme stress).

The WHO-5 is a brief self-assessment for current mental wellbeing.^35^ Respondents rate five items from 0 (‘at no time’) to 5 (‘all of the time’). The raw score (0–25) is multiplied by 5 to give a final score (0–100), where indicates worst and 100 best possible well-being.

### Statistical analysis

The statistical analysis was structured in multiple parts. First, descriptive statistics provided an overview of the mean score per person based on completed (mental) health questionnaires. A detailed analysis of MHQoL dimensions was conducted, reporting mean scores per dimension along with standard errors, medians and minimum-maximum values.

Second, Cronbach’s alpha and McDonald’s omega (ω), along with their 95% CIs were calculated for the seven MHQoL dimensions to assess internal consistency overall and per country. Internal consistency reflects the extent to which test items measure the same construct.^36^ Cronbach’s alpha assumes tau-equivalence, whereas McDonald’s omega provides a more flexible estimate of reliability that does not rely on this assumption. Alpha values range from 0 (no correlation) to 1 (full correlation), with values over 0.70^36^.

Third, to examine construct validity, measuring invariance of the MHQoL across countries was tested using multi-group confirmatory factor analysis (CFA). Invariance was assessed hierarchically^37^ starting with configural invariance (same pattern of factor loadings across groups), followed by metric invariance (statistically identical factor loadings) and scalar invariance (statistically equal thresholds).^38^ Given the ordinal scale, Weighted Least Squares with Mean and Variance adjustments were applied,^38^ using thresholds instead of intercepts while residual invariance was not assessed.^38^ Model fit was evaluated using the Comparative Fit Index (CFI), root mean square error of approximation (RMSEA) and standardised root mean square residual (SRMR), with invariance thresholds set at CFI≥0.95, RMSEA≤0.06 and SRMR≤0.08.^39^ A χ² difference test was conducted to assess significant differences in model fit between levels of invariance. Besides, Spearman’s rank correlation coefficients were calculated to assess associations between: MHQoL and MHQoL VAS; EQ-5D-5L preference-based utilities and LSS with MHQoL and MHQoL VAS at both overall and country levels. Spearman’s rank correlation was chosen over Pearson’s R due to the ordinal nature of MHQoL dimensions.^40^ The correlation (Spearman’s r) between EQ-5D-5L and MHQoL scores was expected to be negative, as better (mental) QoL results in lower EQ-5D-5L sum scores but higher MHQoL scores. Conversely, EQ-5D-5L utilities were expected to show positive correlations, as higher utility scores indicate better QoL. Lastly, two multilevel regression analyses were conducted to account for the hierarchical structure of the data, with individuals nested within countries. The first analysis examined the association between MHQoL dimensions and MHQoL-VAS, while the second assessed the impact of EQ-5D-5L dimensions on MHQoL to identify health-related QoL factors influencing mental QoL. Both models included fixed effects for predictors, along with individual-level covariates (age and gender (in this study the term gender is used to refer to sex assigned at birth). Two model specifications were compared: (1) a random intercept model, allowing intercepts to vary across countries and (2) a random intercept and random slope model, allowing both intercepts and slopes to vary. A likelihood ratio test determined the best-fitting model, favouring the random intercept model. Model fit was evaluated using adjusted R^2^, ranging from 0 (no fit) to 1 (perfect fit).^41^

To evaluate convergent validity, Spearman’s rank correlation coefficients were calculated between the MHQoL and disease-specific mental health questionnaires (eg, PHQ-9, GAD-7, ISI, PSS-4, PRS, WHO-5), and likewise between the EQ-5D-5L and these questionnaires.

P-values were calculated, with significance set at p<0.001. Spearman’s rank correlation coefficients (Spearman’s r) were classified as very strong (0.90–1.00), strong (0.70–0.89), moderate (0.40–0.69), weak (0.10–0.39) and negligible (0.0–0.10).^42^

All the statistical analyses were performed using R V.4.3.2.^43^

### Development of the standardised MHQoL toolbox

The standardised MHQoL toolbox for transformation, calculation, and presentation of collected MHQoL responses was developed as both an R-package and R-Shiny application. The package allows for standardised data transformations and calculations by providing a set of functions in R. It also forms a foundational basis of the application and is based on the original MHQoL manual together with the Dutch value set by van Krugten *et al*.^19 44^

The R-Shiny application was built as an interactive tool to analyse, interpret, and visualise (up)loaded user data. The aim was to provide a graphical user interface for accessible result generation and presentation without the need for deeper knowledge of the statistical programming language R.

The application uses transformation functions from the R-package to convert, test, and score individual responses into scores and utility values, including reverse transformations. Calculation functions are implemented to compute both individual and average LSS and utility values for each respondent automatically.

Throughout development, one author documented all steps and implemented the function, after which a second author independently verified the outputs and usability of the toolbox by reproducing the calculations and checking the consistency of results across example datasets. This verification involved comparing the toolbox outputs with the results obtained to ensure that the implemented functions reproduced the expected results.

A list of R packages used both for the statistical analyses and in the development of the toolbox can be found in online supplemental appendix B.

### Patient and public involvement

Members of the public were involved in the development and design of the study, including the selection of relevant outcomes and the overall approach.^22^ However, they were not involved in the conduct of the analyses or interpretation of the results presented in this paper.

## Results

### Descriptives

In general, all participants scored low on stress, anxiety, depression and insomnia severity scales. Health-related QoL (EQ-5D-5L) showed favourable utility and LSS outcomes across all countries, with preference- based utilities aligning with expected values for a healthy population.^45^ While MHQoL and MHQoL-VAS scores did not indicate poor mental QoL, they suggested more room for improvement than EQ-5D-5L. The UK consistently reported higher problem severity across all questionnaires. No country differences were found in MHQoL dimension-specific scores. All mean scores for (mental) health questionnaires and MHQoL dimensions are detailed in online supplemental tables 2 and 3 of appendix A.

### Internal consistency MHQoL

The MHQoL demonstrated strong internal consistency across all countries, with Cronbach’s α and McDonald’s ω generally exceeding 0.7. While the lower bound of the 95% CI for Cronbach’s α in Finland fell slightly below 0.7, McDonald’s ω remained above 0.7 across all countries. Additionally, a strong correlation was found between the MHQoL score and MHQoL-VAS in all countries (Spearman’s r>0.7, p<0.001), confirming consistency between those two measures. Detailed results are presented in table 1.

**Table 1:** Internal consistency expressed in Cronbach’s alpha of the seven MHQoL dimensions and Spearman’s rank correlation coefficients expressed in Spearman’s r for Finland, Spain, the UK and overall to present the correlation between the MHQoL score and MHQoL VAS

Country	Cronbach’s alpha for the seven MHQoL dimensions*	McDonald’s omega for the seven MHQoL dimensions*	Spearman’s r to present the correlation between the MHQoL and MHQoL VAS
Finland (N=122)	0.741 (0.652 to 0.801)	0.768 (0.703 to 0.820)	0.702†
Spain (N=114)	0.806 (0.710 to 0.859)	0.814 (0.742 to 0.869)	0.767†
UK (N=328)	0.801 (0.761 to 0.835)	0.804 (0.768 to 0.836)	0.771†
Overall (N=564)	0.787 (0.755 to 0.815)	0.796 (0.765 to 0.822)	0.753†

* Coefficient (95% CI).

† p<0.001

MHQoL, Mental Health Quality of Life; VAS, Visual Analogue Scale.

### Construct validity

#### Measurement invariance test

The measurement invariance test (table 2) indicated good fit for the configural model, confirming a consistent factor loading pattern across countries. Metric invariance was supported, suggesting statistically equivalent factor loadings. However, the scalar model showed significant misfit compared with the metric model. To address this, thresholds for several items (Self-Image, Independence, Relationships and Physical Health) were freed, resulting in a partial scalar invariance model with acceptable fit indices. According to established guidelines, partial scalar invariance can still allow meaningful comparisons of latent factor means when a sufficient number of parameters remain invariant across groups.^46^ Therefore, the partial scalar invariance model was retained for cross-country comparisons of the MHQoL. The specific thresholds that were freed are reported in online supplemental table 4 of appendix A.

**Table 2:** Measurement invariance test

Model	CFI	RMSEA (90% CI)	SRMR	Model comparison	Δx^2^ (Δdf)	ΔCFI	ΔRMSEA	ΔSRMR
Model 1: Configural invariance	0.997	0.036 (0.000 to 0.064)	0.058	–	–	–	–	
Model 2: Metric invariance	0.993	0.047 (0.017 to 0.070)	0.070	Model 1	17.347 (12)	−0.004	0.011	0.012
Model 3: Scalar invariance	0.961	0.095 (0.079 to 0.111)	0.065	Model 2	153.76* (20)	−0.032	0.048	−0.005
Model 3a: Partial scalar invariance	0.993	0.033 (0.013 to 0.067)	0.063	Model 2	3.251 (4)	0.000	−0.024	−0.070

* p<0.001.

CFI, Comparative Fit Index; RMSEA, root mean square error of approximation; SRMR, standardised root mean square residual .

#### Relationship between the MHQoL dimensions and VAS score

**Figure 1:**
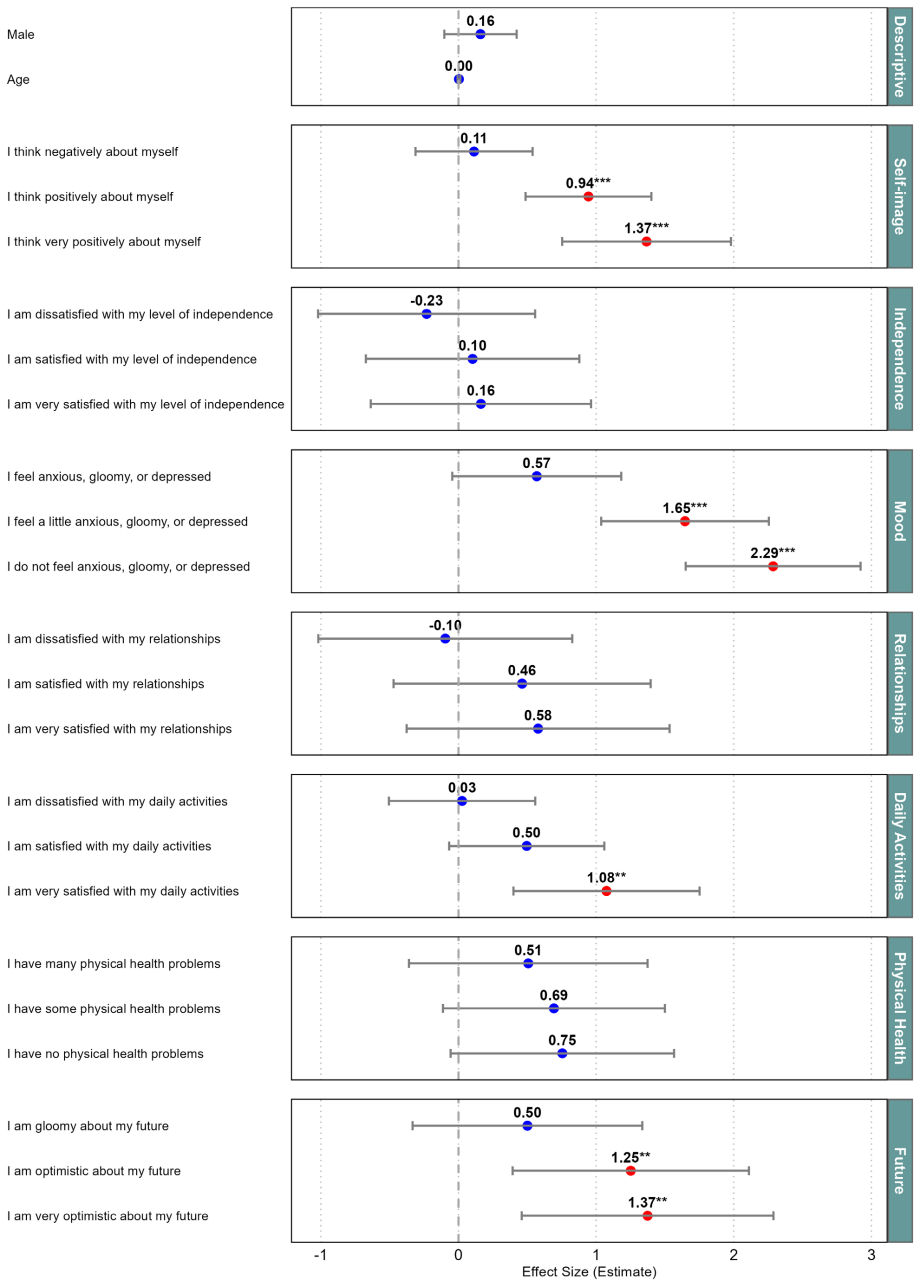
Multilevel regression analysis of the effects of the MHQoL dimensions on the MHQoL VAS score, in which *p<0.05, **p<0.01, ***p<0.001. MHQoL, Mental Health Quality of Life; VAS, Visual Analogue Scale.

Figure 1 presents the multilevel regression analysis examining the influence of MHQoL dimensions on the MHQoL-VAS, with an adjusted R^2^ of 0.641. MHQoL-VAS scores were strongly associated with Self-Image, Daily activities, Mood and Future dimensions. Higher levels generally corresponded to higher VAS coefficients, except for Independence and Relationships, where the second category (‘I am dissatisfied …’) showed a lower coefficient than a more severe level (‘I am very dissatisfied …’). However, these coefficients were not statistically significant, as their CIs crossed zero and extended into both negative and positive values. No significant difference in MHQoL scores was observed between male and female participants, and age was also not significantly associated with MHQoL scores.

#### Correlation between the EQ-5D-5L and MHQoL

Spearman’s r coefficients (table 3) revealed moderate correlations between the MHQoL and both the EQ-5D-5L LSS and preference-based utilities, with stronger correlations for utilities, except in Finland. As expected, correlations with EQ-5D-5L LSS were negative, whereas those with utilities were positive. For the MHQoL VAS, moderate correlations were observed in Spain, the UK and the overall sample across both EQ-5D-5L LSS and utilities, while Finland showed weaker correlations.

**Table 3:** Spearman’s rank correlation coefficients expressed in Spearman’s r for Finland, Spain, the UK and overall to present the correlation between the MHQoL score and MHQoL VAS with the EQ-5D-5L score and EQ-5D-5L utility

Spearman’s r^1^				
Country	**EQ-5D-5L LSS**		**EQ-5D-5L Utility**	
Finland				
MHQoL	−0.565*		0.510*	
MHQoL VAS	−0.332*		0.270	
Spain				
MHQoL	−0.628*		0.687*	
MHQoL VAS	−0.464*		0.528*	
UK				
MHQoL	−0.568*		0.617*	
MHQoL VAS	−0.496*		0.528*	
Overall				
MHQoL	−0.585*		0.617*	
MHQoL VAS	−0.450*		0.554*	

* p value<0.001

EQ-5D-5L, EuroQol 5-Dimension 5-Level questionnaire; LSS, Level Sum Score; MHQoL, Mental Health Quality of Life; VAS, Visual Analogue Scale.

#### Relationship between the EQ-5D dimensions and MHQoL score

**Figure 2:**
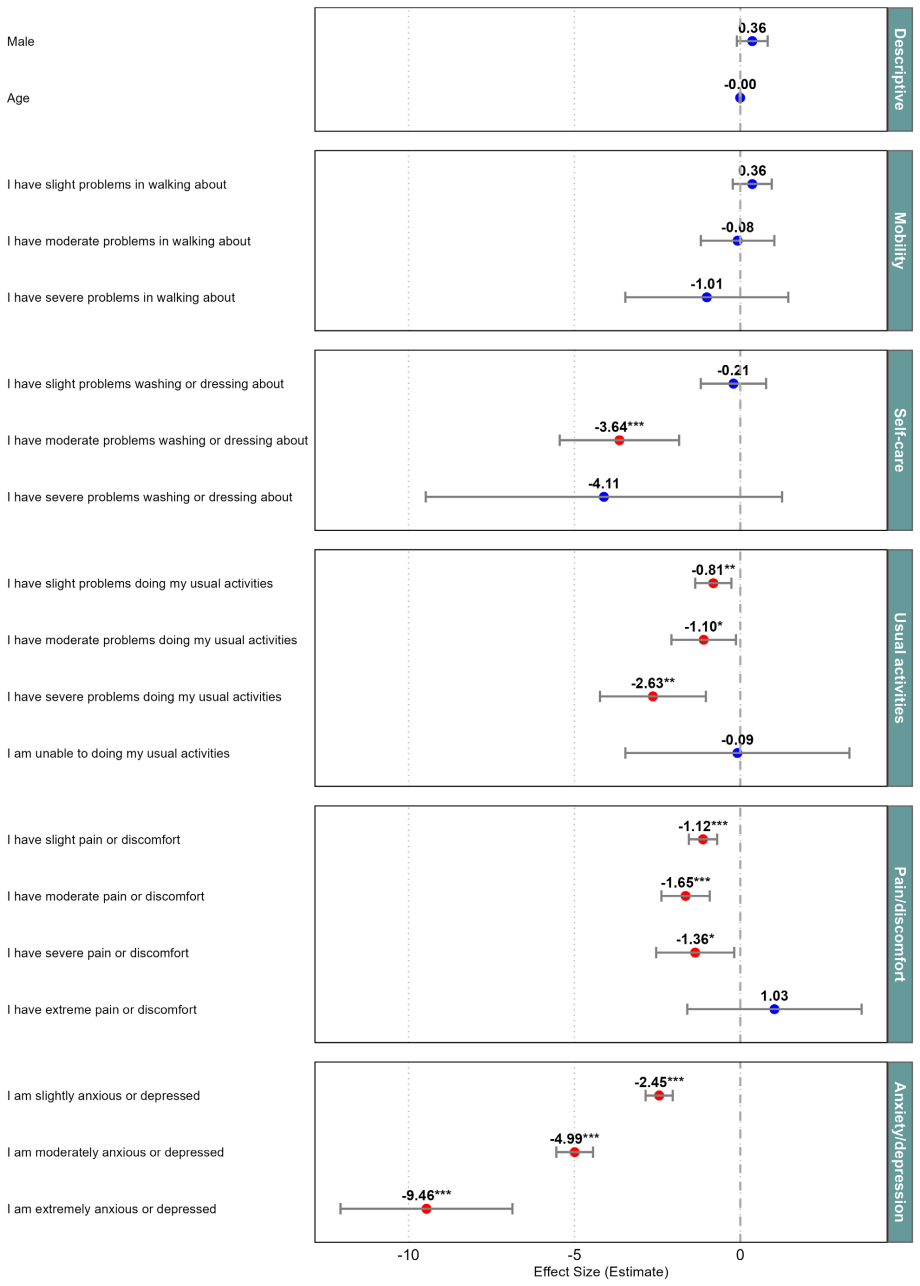
Multilevel regression analysis of the effects of the EQ-5D dimensions on the MHQoL score, in which *p<0.05, **p<0.01, ***p<0.001. EQ-5D, Five-Dimension EuroQol Questionnaire; MHQoL, Mental Health Quality of Life.

The results of the multilevel regression model to examine the influence of the EQ-5D-5L dimensions on the MHQoL score are presented in figure 2, with an adjusted R^2^ of 0.565. As might be expected, the Anxiety/Depression dimension primarily influenced the MHQoL score. Additionally, some levels of the Pain/Discomfort, Usual Activities and Self-care also had a significant impact on the MHQoL score. Neither gender (male vs female) nor age showed a significant association with the MHQoL score.

### Convergent validity

#### Correlations of the EQ-5D-5L and MHQoL with the disease-specific mental health questionnaires

Correlation strengths varied across metrics and regions (table 4). Overall, the MHQoL showed stronger correlations than the EQ-5D-5L in all countries, except for the insomnia questionnaire (ISI) in Spain. The depression (PHQ-9) and well-being (WHO-5) questionnaires exhibited strong correlations with the MHQoL, while anxiety (GAD-7), insomnia (ISI), stress (PSS-4) and work stress (PRS) showed moderate correlations. For the EQ-5D-5L, the PHQ-9, GAD-7, ISI and PSS-4 were moderately correlated, while ISI and PRS showed weak correlations. In the UK, MHQoL correlations with ISI and PRS were weak, whereas EQ-5D-5L correlations followed the overall trend. In Finland, PSS-4 correlated strongly with MHQoL, but most EQ-5D-5L correlations with mental health questionnaires were weak, except for PHQ-9 (moderate). In Spain, all questionnaires except PRS showed moderate correlations with EQ-5D-5L, while MHQoL followed the overall correlation pattern.

**Table 4:** Spearman’s rank correlation coefficients expressed in Spearman’s r for Finland, Spain, the UK and overall to present the correlation between the MHQoL and EQ-5D-5L with the disease-specific mental health questionnaires

Spearman’s r^1^		
Country	MHQoL	EQ-5D-5L
Finland		
PHQ-9	−0.790*	−0.426*
GAD-7	−0.570*	−0.267
ISI	−0.458*	−0.230
PSS-4	−0.719*	−0.275
PRS	−0.502*	−0.385*
WHO-5	0.766*	0.339*
Spain		
PHQ-9	−0.735*	−0.633*
GAD-7	−0.632*	−0.622*
ISI	−0.441*	−0.488*
PSS-4	−0.697*	−0.530*
PRS	−0.530*	−0.363*
WHO-5	0.790*	0.670*
UK		
PHQ-9	−0.727*	−0.586*
GAD-7	−0.607*	−0.446*
ISI	−0.348*	−0.326*
PSS-4	−0.698*	−0.454*
PRS	−0.330*	−0.237*
WHO-5	0.778*	0.573*
Overall		
PHQ-9	−0.750*	−0.585*
GAD-7	−0.605*	−0.491*
ISI	−0.395*	−0.342*
PSS-4	−0.698*	−0.479*
PRS	−0.397*	−0.278*
WHO-5	0.780*	0.589*

* p<0.001.

EQ-5D-5L, EuroQol 5-Dimension 5-Level questionnaire; GAD-7, Generalised Anxiety Disorder-7; ISI, Insomnia Severity Index; MHQoL, Mental Health Quality of Life; PHQ-9, Patient Health Questionnaire-9; PRS, Psychosocial Risk Scale; PSS-4, Perceived Stress Scale-4; WHO-5, World Health Organization-Five Well-Being Index.

### The MHQoL toolbox

Both the MHQoL R-package and the accompanying R-Shiny application are publicly available on CRAN under the names MHQoL^47^ and MHQoL-Toolbox (https://bit.ly/MHQoL-Toolbox), respectively, indicating that it has successfully met rigorous quality standards, including comprehensive functionality testing, compliance with documentation requirements and proper dependency management. As a result, the package and tool are stable, well documented and openly accessible.

Independent verification by two authors (SP and FWT) confirmed that the package and tool consistently produced identical scores to manual MHQoL calculations, ensuring accuracy. Additionally, both calculation and result presentation were completed in under one second, significantly enhancing computation efficiency.

Because the code is publicly accessible on GitHub, a continuous feedback mechanism is established, allowing users to provide input and contribute to ongoing improvements in the user experience and addition of value sets.

## Discussion

### Main results

Our findings indicate a strong and consistent relationship between the MHQoL, MHQoL- VAS and other mental health questionnaires across all participating countries. This supports the robustness of the MHQoL as a measure of mental health QoL. While statistical tests such as Cronbach’s Alpha, McDonald’s Omega, invariance testing and Spearman’s rank correlation confirm these relationships, the primary implication is the reliability and validity of the MHQoL in a working population across different countries and contexts. This study established a solid foundation for the development of a standardised toolbox (R package and Shiny tool) enabling efficient MHQoL calculation, transformation and presentation, thereby streamlining the assessment process and enhancing its ease of use.

More specifically, the MHQoL demonstrated strong correlations not only among its own dimensions but also when compared with other questionnaires and between countries, underscoring its robust internal consistency and construct validity. In a comparative analysis with the EQ-5D-5L and disease-specific mental health questionnaires, the MHQoL consistently showed higher correlations with the disease-specific questionnaires across nearly all measures, although somewhat weaker associations between the MHQoL and other mental health instruments were observed in the Finnish sample. These differences may partly reflect the absence of a country-specific EQ-5D-5L value set for Finland, which required the use of the Danish value set as a proxy and may have influenced the estimated utility-based correlations.

Despite these overall strong associations, not all dimensions appear to be of equal importance. The multilevel analysis highlighted that the dimensions of Self-image, Mood, Daily activities and Future particularly impact the MHQoL VAS and thereby overall mental health QoL. From a broader perspective, it becomes apparent that not only mental health aspects correlate with QoL, but physical aspects do so as well. Specifically, dimensions such as Pain/Discomfort, Usual Activities and Self-care showed significant correlations.

compared with the psychometric studies investigating the properties of the MHQoL in the broader population and other countries, such as the study by Enzing *et al*,^20^ the MHQoL continues to demonstrate similarly strong psychometric properties, both for the internal consistency and construct validity. Although only the influence of the Anxiety/Depression dimension of the EQ-5D-5L on the MHQoL was measured, recommendations were made to further analyse the relationship between the MHQoL and EQ-5D-5L.^20^

Our study demonstrated that the MHQoL appears to have an enhanced sensitivity in assessing mental health QoL compared with the EQ-5D-5L. However, outcomes of both the MHQoL and EQ-5D-5L seem to be interdependent, suggesting that the dimensions of these questionnaires influence each other’s outcomes. This implies that while the MHQoL offers added value in capturing mental health-specific dimensions, it may be most informative when used alongside generic instruments like the EQ-5D-5L. Together, these tools can provide a more comprehensive assessment of both general and mental health-related QoL, which is particularly relevant for economic evaluations and decision-making in health policy and workplace interventions.

### Strengths and limitations

A key strength of this study was the inclusion of a multi-country dataset, enabling cross-national comparisons and accounting for cultural differences in work environments. Additionally, reliability and validity were assessed using multiple approaches. Unlike prior studies focused solely on the Anxiety/Depression dimension of the EQ-5D-5L, this study conducted additional analyses to explore broader correlations. Furthermore, the development of a tool streamlined the transformation, calculation and presentation of the MHQoL results, enhancing usability and reproducibility in this and future studies. Importantly, this study was among the first to evaluate the MHQoL in a working population, providing critical insights for economic evaluations in occupational health research. Finally, the combination of psychometric validation with applied tool development supports both theoretical and practical advancement in the measurement of mental health-related QoL.

However, several limitations should be acknowledged. First, the sample included very few blue-collar employees, was predominantly female and highly educated, and largely consisted of public sector workers from three sectors that varied between countries. This may limit the generalisability of findings to the broader population. In addition, the voluntary nature of participation may have introduced selection bias, as employees with a greater interest in mental health may have been more likely to participate, which may be reflected in the observed sample. Second, most data originated from the UK, making the overall results less representative of Spain and Finland. Additionally, the absence of a country-specific value set for Finland’s EQ-5D-5L preference-based utilities required the use of a proxy, potentially affecting the accuracy of correlations. Lastly, due to the relatively small sample size, certain EQ-5D-5L dimensions had low or no responses, leading to coefficients with high uncertainty or, in some cases, the inability to estimate coefficients. Furthermore, the analyses were based only on cross-sectional baseline data. Although the broader trial included follow-up measurements, these contained substantial missing data, which prevented reliable assessment of test-retest reliability. As a result, the present study could not evaluate temporal stability or responsiveness, and construct validation was limited to static cross-sectional patterns. In addition, the analyses relied primarily on self-reported measures (eg, MHQoL, MHQoL VAS and EQ-5D-5L). As a result, the observed associations may partly reflect common method variance, as responses were obtained using similar measurement approaches at the same time point. This may have contributed to stronger correlations between measures and should be considered when interpreting the results. Finally, a limitation of this study is that, to date, a value set for the MHQoL is only available for the Netherlands,^44^ which restricts its immediate use in economic evaluations across other countries. However, the developed tool is designed to be adaptable and will support the integration of additional country-specific value sets as they become available, facilitating future international applications.

### Recommendations and implications

The findings from this study, along with previous research,^19 20^ demonstrate that the MHQoL is a valid and reliable questionnaire for measuring mental health QoL both in the general and working populations. Since all validation studies with MHQoL have been performed in Western Europe, extension to other regions would give more insight into the global applicability of this instrument. Furthermore, research in other groups of workers such as those in white-collar occupations, a higher proportion of men and individuals employed in business environments would provide a more comprehensive understanding of the working population. Moreover, gathering more disease-specific data is crucial. To achieve this, it is essential to incorporate the MHQoL in studies of mental health problems wherever possible.

In addition, this study provides preliminary evidence suggesting that the MHQoL may better capture the domains of mental health issues than the EQ-5D-5L. Further research is necessary to fully confirm this, both in the general and in the working population. If further evidence supports the positive outcomes observed, the MHQoL should be utilised to measure QoL in mental health issues alongside the EQ-5D-5L. This is because the questionnaires appear to influence each other’s outcomes. Consequently, the relationship between the two questionnaires can be better examined, and a more comprehensive picture of the QoL can be depicted. However, for the application of the MHQoL in economic evaluations, data on the utilities of various dimensions are required from multiple countries; as of now, such data is available only for the Netherlands.^44^

## Conclusion

This study demonstrated that the MHQoL was a reliable and valid instrument for assessing mental health-related QoL in working populations across Finland, Spain and the United Kingdom. By focusing on employed individuals, this study also contributed to the growing field of economic evaluations targeting workplace mental health interventions, a domain of increasing societal and policy relevance due to the substantial burden of mental health problems on productivity, absenteeism and healthcare costs.

The results suggested that the MHQoL showed enhanced sensitivity to mental health domains compared with general preference-based instruments such as the EQ-5D-5L and SF-36. Importantly, the MHQoL added value when used alongside these instruments in economic evaluations, by capturing aspects of QoL more directly related to mental well-being. Furthermore, a standardised and accessible toolbox, including an open-source R package and Shiny application, was developed to support the consistent scoring, transformation and reporting of MHQoL data, promoting broader application in both research and practice.

The use of a sensitive and targeted instrument like the MHQoL can help ensure that future evaluations more accurately capture the impact of mental health interventions in occupational settings.

## Supplementary material

10.1136/bmjopen-2025-108598 online supplemental file 1

10.1136/bmjopen-2025-108598 online supplemental file 2

## Data Availability

Data are available on reasonable request.
