# Supplementary material for: Cross-national validation of the MHQoL: psychometric evaluation and open-source tools for assessing mental health quality of life
Source: BMJ Open. 2026 May 13;16(5):e108598. doi: 10.1136/bmjopen-2025-108598 (PMC13182401; doi:10.1136/bmjopen-2025-108598)
Supplement: online supplemental file 1 [file bmjopen-16-5-s001.docx]

# **Appendix A – Tables**

**Table 1:** General personal and employment characteristics of the included participants

| Characteristic | Finland^1^  (N = 122) | Spain^1^  (N = 114) | United Kingdom^1^  (N= 328) | Overall^1^  (N = 564) |
| --- | --- | --- | --- | --- |
| Age | 46.5 (10.5) | 47.8 (9.7) | 43.1 (10.5) | 44.8 (10.5) |
| Sex at birth | | | | |
| Female | 111 (91.0%) | 64 (56.1%) | 272 (82.9%) | 447 (79.3%) |
| Male | 11 (9.0%) | 50 (43.9%) | 53 (16.2%) | 114 (20.2%) |
| Unknown | 0 (0%) | 0 (0%) | 3 (0.9%) | 3 (0.5%) |
| Education | | | | |
| Primary school completed | 0 (0%) | 2 (1.8%) | 0 (0%) | 2 (0.4%) |
| Some secondary school – not completed | 2 (1.6%) | 3 (2.6%) | 0 (0%) | 5 (0.9%) |
| Secondary school - vocational | 25 (20.5%) | 15 (13.2%) | 3 (0.9%) | 43 (7.6%) |
| GCSE | 37 (30.3%) | 32 (28.1%) | 23 (7.0%) | 92 (16.3%) |
| A-levels | 52 (42.6%) | 36 (31.6%) | 27 (8.2%) | 115 (20.4%) |
| University degree completed | 6 (4.9%) | 26 (22.8%) | 275 (83.8%) | 307 (54.4%) |
| Working hours | 36.3 (9.16) | 37.4 (6.43) | 35.1 (6.20) | 35.8 (7.03) |
| Designation | | | | |
| Blue Collar | 10 (8.2%) | 9 (7.9%) | 3 (0.9%) | 22 (3.9%) |
| White Collar | 112 (91.8%) | 102 (89.5%) | 325 (99.1%) | 539 (95.6%) |
| Unknown | 0 (0%) | 3 (2.6%) | 0 (0%) | 3 (0.5%) |
| Company type | | | | |
| Large company | 0 (0%) | 9 (7.9%) | 0 (0%) | 9 (1.6%) |
| Public agency | 122 (100%) | 37 (32.5%) | 328 (100%) | 487 (86.3%) |
| SME | 0 (0%) | 68 (59.6%) | 0 (0%) | 68 (12.1%) |
| Economic sector | | | | |
| Education & training | 0 (0%) | 0 (0%) | 56 (17.1%) | 56 (9.9%) |
| Health and life science | 0 (0%) | 0 (0%) | 272 (82.9%) | 272 (48.2%) |
| Manufacturing industry | 0 (0%) | 68 (59.6%) | 0 (0%) | 68 (12.1%) |
| Public administration | 122 (100%) | 37 (32.5%) | 0 (0%) | 159 (28.2%) |
| Real estate | 0 (0%) | 2 (1.8%) | 0 (0%) | 2 (0.4%) |
| Support services | 0 (0%) | 7 (6.1%) | 0 (0%) | 7 (1.2%) |

**Table 2**: Mean (standard deviation)) scores of the (mental) health related questionnaires for Finland, Spain the United Kingdom and overall

| (Mental) Health Questionnaires | Finland^1^  N = 122 | Spain^1^  N = 114 | United Kingdom^1^  N = 328 | Overall^1^  N = 564 |
| --- | --- | --- | --- | --- |
| Depression (PHQ-9) | 6.35 (4.96) | 5.67 (5.07) | 7.97 (5.91) | 7.15 (5.63) |
| Anxiety (GAD7) | 4.67 (4.24) | 5.68 (4.67) | 6.60 (5.27) | 6.0 (5.0) |
| Insomnia (ISI) | 3.36 (2.54) | 3.45 (2.82) | 3.62 (2.60) | 3.53 (2.63) |
| General Stress (PSS4) | 4.64 (2.85) | 4.76 (2.93) | 6.80 (3.16) | 5.92 (3.22) |
| Work Stress (PRS) | 10.0 (8.20) | 9.13 (9.09) | 10.8 (10.1) | 10.3 (9.52) |
| Wellbeing (WHO5) | 56.7 (18.3) | 56.7 (20.8) | 44.9 (21.2) | 49.8 (21.3) |
| MHQoL | 14.4 (2.79) | 14.7 (3.18) | 13.5 (3.44) | 13.9 (3.29) |
| MHQoL VAS | 7.18 (1.84) | 6.83 (1.84) | 6.33 (1.92) | 6.62 (1.92) |
| EQ-5D-5L LSS | 8.98 (2.24) | 8.47 (2.72) | 9.49 (3.35) | 9.18 (3.04) |
| EQ-5D-5L VAS | 64.8 (27.1) | 74.0 (20.4) | 71.7 (20.1) | 70.6 (22.0) |
| EQ5D utility | 0.919 (0.088) | 0.882 (0.128) | 0.847 (0.143) | 0.870 (0.133) |

**Table 3**: Mean and median scores of the MHQoL dimensions for Finland, Spain the United Kingdom and overall

| Characteristic | Finland  N = 122 | Spain  N = 114 | United Kingdom  N = 328 | Overall  N = 564 |
| --- | --- | --- | --- | --- |
| MHQoL Dimensions | | | | |
| Self-image |  |  |  |  |
| *Mean (SD)* | 1.96 (0.581) | 1.97 (0.572) | 1.41 (0.792) | 1.64 (0.761) |
| *Median [Min, Max]* | 2.0 [0, 3.0] | 2.0 [0, 3.0] | 1.0 [0, 3.0] | 2.0 [0, 3.0] |
| Independence |  |  |  |  |
| *Mean (SD)* | 2.06 (0.696) | 2.11 (0.713) | 2.22 (0.691) | 2.16 (0.699) |
| *Median [Min, Max]* | 2.0 [0, 3.0] | 2.0 [0, 3.0] | 2.0 [0, 3.0] | 2.0 [0, 3.0] |
| Mood |  |  |  |  |
| *Mean (SD)* | 2.30 (0.757) | 2.35 (0.704) | 2.01 (0.809) | 2.14 (0.792) |
| *Median [Min, Max]* | 2.0 [0, 3.0] | 2.0 [1.0, 3.0] | 2.0 [0, 3.0] | 2.0 [0, 3.0] |
| Relationships |  |  |  |  |
| *Mean (SD)* | 2.14 (0.621) | 2.05 (0.727) | 2.13 (0.728) | 2.12 (0.706) |
| *Median [Min, Max]* | 2.0 [1.0, 3.0] | 2.0 [0, 3.0] | 2.0 [0, 3.0] | 2.0 [0, 3.0] |
| Daily activities |  |  |  |  |
| *Mean (SD)* | 1.80 (0.651) | 1.80 (0.681) | 1.61 (0.717) | 1.69 (0.702) |
| *Median [Min, Max]* | 2.0 [0, 3.0] | 2.0 [0, 3.0] | 2.0 [0, 3.0] | 2.0 [0, 3.0] |
| Physical health |  |  |  |  |
| *Mean (SD)* | 2.07 (0.645) | 2.40 (0.725) | 2.27 (0.661) | 2.26 (0.679) |
| *Median [Min, Max]* | 2.0 [0, 3.0] | 3.0 [0, 3.0] | 2.0 [0, 3.0] | 2.0 [0, 3.0] |
| Future |  |  |  |  |
| *Mean (SD)* | 2.03 (0.462) | 1.96 (0.531) | 1.87 (0.688) | 1.93 (0.618) |
| *Median [Min, Max]* | 2.0 [1.0, 3.0] | 2.0 [0, 3.0] | 2.0 [0, 3.0] | 2.0 [0, 3.0] |

| MHQoL domain | Response-category boundary allowed to vary across countries |
| --- | --- |
| Self-image | I think negatively about myself ↔ I think negatively about myself  I think negatively about myself ↔ I think positively about myself  I think positively about myself ↔ I think very positively about myself |
| Independence | I am very dissatisfied with my level of independence ↔ I am dissatisfied with my level of independence  I am dissatisfied with my level of independence ↔ I am satisfied with my level of independence |
| Relationships | I am satisfied with my relationships ↔ I am very satisfied with my relationships |
| Physical health | I have some physical health problems ↔ I have no physical health problems |

Table 4: Response-category thresholds freed in the partial sclar invariance model
