# Supplementary material for: Cross-national validation of the MHQoL: psychometric evaluation and open-source tools for assessing mental health quality of life
Source: BMJ Open. 2026 May 13;16(5):e108598. doi: 10.1136/bmjopen-2025-108598 (PMC13182401; doi:10.1136/bmjopen-2025-108598)
Supplement: online supplemental file 2 [file bmjopen-16-5-s002.docx]

# **Appendix B - Packages**

## **eq5d**

The eq5d package offers tools for analyzing EQ-5D profiles both at a single point in time and over periods, as well as for calculating index scores based on an individual’s dimension scores ^1^. This package was used for the calculation of the EQ-5D preference-based utilities.

## **emmeans**

Functions to calculate estimated marginal means (EMMs) for various types of models, including linear, generalized linear and mixed models ^2^. Used in this study for the calculation of marginal mans of the generalized linear models

**lavaan**

The lavaan package provides a comprehensive framework for estimating a wide range of multivariate statistical models, such as path analysis, confirmatory factor analysis, structural equation models, and latent growth curve models . In this study, it was used specifically to perform a confirmatory factor analysis.^3^

## **table1**

The table 1 package provides tools for creating descriptive tables, which were utilized in this study for that purpose ^4^.

## **tidyverse**

This package is designed to simplify the installation and loading of multiple tidyverse packages in a single step ^5^. In this study, it was also used in this capacity.

**References**

1. Morton F, Nijjar JS. eq5d: Methods for Analysing “EQ-5D” Data and Calculating “EQ-5D” Index Scores. Published online September 4, 2024. https://github.com/fragla/eq5d

2. Lenth RV. emmeans: Estimated Marginal Means, aka Least-Squares Means. Published online October 14, 2024. https://rvlenth.github.io/emmeans/,https://rvlenth.github.io/emmeans/

3. Rosseel Y. **lavaan** : An *R* Package for Structural Equation Modeling. *J Stat Soft*. 2012;48(2). doi:10.18637/jss.v048.i02

4. Rich B. table1: Tables of Descriptive Statistics in HTML. Published online January 6, 2023. https://github.com/benjaminrich/table1

5. Wickham H. tidyverse: Easily Install and Load the “Tidyverse.” Published online February 22, 2023. https://github.com/tidyverse/tidyverse
