# Supplementary material for: Cross-national validation of the MHQoL: psychometric evaluation and open-source tools for assessing mental health quality of life
Source: BMJ Open. 2026 May 13;16(5):e108598. doi: 10.1136/bmjopen-2025-108598 (PMC13182401; doi:10.1136/bmjopen-2025-108598)
Supplement: Supplementary file 3 [file bmjopen-2025-108598.reviewer_comments.pdf]

PEER REVIEW HISTORY

BMJ Open publishes all reviews undertaken for accepted manuscripts. Reviewers are asked to complete a checklist review form and are provided with free text boxes to elaborate on their assessment. These free text comments are reproduced below.

ARTICLE DETAILS

Title (Provisional)

Cross-National Validation of the MHQoL: Psychometric Evaluation and Open-Source Tools for Assessing Mental Health Quality of Life

Authors

Peeters, Stijn; Thielen, Frederick W.; de Mul, Marleen; Sinokki, Marjo; Olaya, Beatriz; van der Feltz-Cornelis, Christina Maria; Hakkaart-van Roijen, Leona

---

VERSION 1 - REVIEW

**Reviewer**	**1**
**Name**	**Cwik, Jan**
**Affiliation**	**Universität zu Köln, Clinical Psychology and Psychotherapy**
**Date**	**25-Nov-2025**
**COI**	**None**

This manuscript presents a cross-national psychometric evaluation of the MHQoL instrument and the development of an open-source scoring toolbox using baseline data from the EMPOWER trial across Finland, Spain, and the UK. The topic is timely, relevant, and directly valuable for researchers and practitioners interested in mental health–related quality-of-life assessment. The paper is clearly written and generally well structured. The development of an open-source tool is a major practical contribution.

This is a strong and valuable contribution to the psychometric literature on mental health quality-of-life instruments. With revisions addressing the following methodological issues, the manuscript would be suitable for publication.

The writing is clear and professional; no major language revisions are needed. However, several methodological aspects require clarification or strengthening, particularly regarding (1) sample representativeness, (2) psychometric methods, (3) measurement invariance, (4) limitations of internal consistency reporting, and (5) interpretability of cross-country comparisons.

Major comments:

Below, I outline major and minor methodological concerns.

• Although the total sample size is adequate (N=564), it is highly unbalanced across countries (Finland: n=122; Spain: n=114; UK: n=328). This has multiple implications:

o The UK sample dominates the total model estimates, potentially biasing cross-national comparisons.

o The sample is heavily skewed toward female, highly educated, public-sector, and white-collar workers, limiting generalisability to broader working populations.

o Finland and Spain contribute comparatively small samples, reducing power for reliable country-level psychometric testing.

In the limitations section, the authors acknowledge some of these issues, but the implications for measurement invariance and cross-country comparisons warrant further discussion.

• The authors report Cronbach's α as a reliability metric, but it is not a measure of test reliability; it is a measure of internal consistency, and it is valid only under restrictive assumptions (tau-equivalence). Given that MHQoL items measure distinct conceptual domains (self-image, mood, daily activities, physical health, etc.), the assumption of unidimensionality and tau-equivalence is questionable. Accordingly, I would recommend that the authors report McDonald's ω (omega total or omega hierarchical), which is better suited for scales with heterogeneous indicators.

• Furthermore, please justify treating the MHQoL as internally consistent enough to compute a single α across seven distinct life domains.

• The invariance analysis is well motivated but raises several methodological issues:

o The authors report that scalar invariance failed and only partial scalar invariance could be established by freeing multiple thresholds.

o Partial invariance allows comparisons, but only under rigorous conditions - particularly when multiple thresholds across multiple items need to be freed, interpretability becomes limited.

 I would recommend that the authors provide a more apparent justification for why partial scalar invariance is considered acceptable here. Furthermore, please explicitly state that mean comparisons across countries must be interpreted with caution. And finally, regarding this point, please include a table or figure that identifies the exact parameters freed and discuss the substantive implications (e.g., cultural differences in interpreting self-image or independence items).

• The multilevel regression of MHQoL dimensions on the VAS shows unexpected, non-monotonic patterns - for example, the second category ("I am dissatisfied…") sometimes shows a lower coefficient than more severe categories. Possible issues could include how categories were dummy-coded, cultural or linguistic differences in how items are

interpreted, or nonlinear relationships that require alternative modelling (e.g., ordinal regression or spline functions). The authors should re-examine these patterns and discuss potential explanations.

• Because no Finnish EQ-5D-5L value set existed, the Danish set was used as a proxy. While this is common practice, it introduces limitations because Nordic similarity cannot simply be assumed, and the utility values may be systematically biased. Accordingly, the authors should add a sensitivity analysis (e.g., comparing LSS correlations to utilities) and discuss how this limitation may have contributed to the weaker correlations observed in Finland.

• The study uses cross-sectional baseline data only, which prevents evaluation of test–retest reliability, precludes assessment of responsiveness, and limits construct validation to static patterns, unable to examine temporal stability or sensitivity to change. This limitation should be more explicitly acknowledged, especially since MHQoL is intended for use in economic evaluations, which rely on change scores.

• The description of the R toolbox development is valuable. Still, the validation process is briefly described (“two authors verified the outputs”), and no quantitative validation (e.g., unit tests, error rate assessment, inter-version comparisons) is mentioned. A stronger validation report would enhance the tool's credibility for public use.

Major comments:

• For the CFA model fit index, the RMSEA should be reported with 90%-CI.

• The manuscript relies heavily on Spearman’s correlations; a discussion of potential common method bias (all self-report instruments) would be appropriate.

• Some correlations between mental health instruments and MHQoL appear weaker than expected in Finland; the authors could explore cultural or translation-related factors.

• Figures (e.g., regression plots) would benefit from confidence intervals to enhance interpretability.

**Reviewer**	**2**
**Name**	**Fischer, Simone**
**Affiliation**	**University of Augsburg, Epidemiology**
**Date**	**23-Feb-2026**
**COI**	**None**

Overall, the study addresses an important topic and uses a variety of methods to validate the questionnaire in this workplace sample from three countries.

However, I have a few comments:

1) In the abstract, the results should be more concisely reported in the results section and not repeat the methods. Measurement invariance testing does only make sense if you report the groups that were tested (in this case countries). Please report the groups in the abstract. Please also report the concrete (range of) Cronbach's alpha instead of the threshold in the results section.

2) Authors report that validated versions in Finnish, Spanish and English were used. If so, they should also cite the validation studies of the related versions in the methods section accordingly.

3) Please report the minimum and maximum age of the participants for each sample.

4) The authors state "Gender did have an influence on the MHQoL score" and refer to Figure 2. Please report correctly to which gender the coefficient applies (or add the reference category) to make this information interpretable for the reader. This aspect should also be shortly discussed and compared to other studies on gender differences in mental quality of life.

5) The authors describe limited generalizability due to mostly white-collar, female and higher educated participants. While that is certainly true, there may also be a selection bias. Descriptive statistics e.g. show low scores in anxiety, depression, etc.

The authors should provide information about the sampling strategy of the original study. Since the original study contained an intervention, this could also have had impact on motivation to participate in the study. Even though the details may be described in the study protocol of the original study, a few more information about the sample (recruitment, response) should be provided in this study in the methods and limitation section (selection bias).

6) Baseline data from an intervention study were analyzed. Were follow-up data available to the authors to investigate test-retest reliability or responsiveness/sensitivity to change in mental quality of life? This would strengthen the results regarding psychometric validity in this study or could at least be mentioned for future investigations.

VERSION 1 - AUTHOR RESPONSE

Reviewer 1

1. *Although the total sample size is adequate (N=564), it is highly unbalanced across countries (Finland: n=122; Spain: n=114; UK: n=328). This has multiple implications:*
   o The UK sample dominates the total model estimates, potentially biasing cross-national comparisons.
   o The sample is heavily skewed toward female, highly educated, public-sector, and white-collar workers, limiting generalisability to broader working populations.

o Finland and Spain contribute comparatively small samples, reducing power for reliable country-level psychometric testing.

In the limitations section, the authors acknowledge some of these issues, but the implications for measurement invariance and cross-country comparisons warrant further discussion.

• The authors report Cronbach's α as a reliability metric, but it is not a measure of test reliability; it is a measure of internal consistency, and it is valid only under restrictive assumptions (tau-equivalence). Given that MHQoL items measure distinct conceptual domains (self-image, mood, daily activities, physical health, etc.), the assumption of unidimensionality and tau-equivalence is questionable. Accordingly, I would recommend that the authors report McDonald's ω (omega total or omega hierarchical), which is better suited for scales with heterogeneous indicators.

We agree that Cronbach's α is a measure of internal consistency rather than a direct measure of test reliability. In the manuscript we did not refer to Cronbach's α as a reliability measure but explicitly described it as an indicator of internal consistency. To further clarify this point, we have revised the wording in Section 3.2 and replaced the term constancy with consistency. In line with the reviewer's suggestion, we have now additionally reported McDonald's ω (omega), along with its 95% confidence intervals, as a complementary measure of internal consistency. As noted, Cronbach's α is based on the assumption of tau-equivalence, whereas McDonald's ω provides a more flexible estimate that does not rely on this assumption and is therefore particularly suitable for instruments with heterogeneous indicators such as the MHQoL. At the same time, we retained Cronbach's α to ensure comparability with previous validation studies of the MHQoL, including the original development study and subsequent psychometric evaluations (see The Mental Health Quality of Life Questionnaire (MHQoL): development and first psychometric evaluation of a new measure to assess quality of life in people with mental health problems | Quality of Life Research | Springer Nature Link and Psychometric evaluation of the Mental Health Quality of Life (MHQoL) instrument in seven European countries | Health and Quality of Life Outcomes | Springer Nature Link). Using both indices allows for consistency with the existing literature while providing a more robust assessment of internal consistency.

2. *Furthermore, please justify treating the MHQoL as internally consistent enough to compute a single α across seven distinct life domains.*

We acknowledge that the MHQoL includes items covering several life domains (e.g., self-image, mood, daily activities, and physical health). The decision to calculate a single Cronbach's α across the seven items is based on the conceptualization and psychometric evaluation of the MHQoL in earlier validation studies. Previous studies examining the

measurement properties of the MHQoL, including the original instrument development paper (see The Mental Health Quality of Life Questionnaire (MHQoL): development and first psychometric evaluation of a new measure to assess quality of life in people with mental health problems | Quality of Life Research | Springer Nature Link and Psychometric evaluation of the Mental Health Quality of Life (MHQoL) instrument in seven European countries | Health and Quality of Life Outcomes | Springer Nature Link) evaluated the scale structure and supported the treatment of the items as indicators of an overarching construct of mental health-related quality of life. These studies also reported Cronbach's α for the overall scale, demonstrating adequate internal consistency. Using the same approach in the present study facilitates comparison with earlier validation studies of the instrument.

3. *The invariance analysis is well motivated but raises several methodological issues:*
   o The authors report that scalar invariance failed and only partial scalar invariance could be established by freeing multiple thresholds.
   o Partial invariance allows comparisons, but only under rigorous conditions - particularly when multiple thresholds across multiple items need to be freed, interpretability becomes limited.
   ◊ I would recommend that the authors provide a more apparent justification for why partial scalar invariance is considered acceptable here. Furthermore, please explicitly state that mean comparisons across countries must be interpreted with caution. And finally, regarding this point, please include a table or figure that identifies the exact parameters freed and discuss the substantive implications (e.g., cultural differences in
   interpreting self-image or independence items).

We agree that additional clarification regarding the partial scalar invariance model improves the transparency and interpretation of the analysis. In the revised manuscript, we have expanded the description of the measurement invariance results. Specifically, we now provide a clearer justification for accepting partial scalar invariance and explicitly state that cross-country comparisons should be interpreted with caution because several item thresholds differed between countries. In line with methodological guidelines, we note that partial scalar invariance still allows meaningful comparisons of latent factor means when a sufficient number of parameters remain invariant across groups. Furthermore, as suggested by the reviewer, we have added an additional table in Appendix A (Table X) that lists the exact response-category thresholds that were freed to obtain the partial scalar invariance model. This table improves transparency by identifying which MHQoL items contributed to the lack of full scalar invariance.

4. *The multilevel regression of MHQoL dimensions on the VAS shows unexpected, non-monotonic patterns - for example, the second category ("I am dissatisfied…")*

sometimes shows a lower coefficient than more severe categories. Possible issues could include how categories were dummy-coded, cultural or linguistic differences in how items are interpreted, or nonlinear relationships that require alternative modelling (e.g., ordinal regression or spline functions). The authors should re-examine these patterns and discuss potential explanations.

We re-examined the regression coefficients and their corresponding confidence intervals. Although some coefficients appear non-monotonic (e.g., the second response category occasionally showing a lower coefficient than a more severe category), these coefficients were not statistically significant, and their confidence intervals crossed zero and extended into both negative and positive values. This indicates substantial uncertainty in these estimates. To clarify this point for readers, we have revised the Results section to explicitly report that these coefficients were not statistically significant and that their confidence intervals crossed zero. No changes were made to the modelling approach, as the observed pattern does not indicate a systematic or statistically meaningful deviation.

5. *Because no Finnish EQ-5D-5L value set existed, the Danish set was used as a proxy. While this is common practice, it introduces limitations because Nordic similarity cannot simply be assumed, and the utility values may be systematically biased. Accordingly, the authors should add a sensitivity analysis (e.g., comparing LSS correlations to utilities) and discuss how this limitation may have contributed to the weaker correlations observed in Finland.*

Using a proxy value set may indeed introduce limitations when calculating EQ-5D-5L utilities for Finland. This limitation has already been acknowledged in the Discussion section, where we note that the absence of a country-specific Finnish value set required the use of the Danish value set as a proxy, which may affect the accuracy of the correlations. In addition, Table 3 already reports Spearman's rank correlations for both the EQ-5D-5L utility scores and the EQ-5D-5L level sum score (LSS). Presenting both measures allows readers to compare associations based on the preference-weighted utilities and the non-preference-weighted LSS. Because the LSS does not rely on a country-specific value set, this comparison provides an indication of whether the use of the Danish value set substantially affects the observed correlations.

6. *The study uses cross-sectional baseline data only, which prevents evaluation of test–retest reliability, precludes assessment of responsiveness, and limits construct validation to static patterns, unable to examine temporal stability or sensitivity to change. This limitation should be more explicitly acknowledged, especially since MHQoL is intended for use in economic evaluations, which rely on change scores.*

To clarify this point, we have expanded the limitations section to explicitly state that the present analyses were based only on baseline data. Although the broader trial included

follow-up measurements, these contained substantial missing data, which prevented reliable test–retest analyses. We now explicitly acknowledge that this design precludes the assessment of test–retest reliability, responsiveness, and temporal stability, and that construct validation in the current study is therefore limited to cross-sectional patterns. These clarifications have been added to the limitations section of the Discussion.

7. *The description of the R toolbox development is valuable. Still, the validation process is briefly described ("two authors verified the outputs"), and no quantitative validation (e.g., unit tests, error rate assessment, inter-version comparisons) is mentioned. A stronger validation report would enhance the tool's credibility for public use.*

The toolbox does not implement new statistical estimation procedures but primarily facilitates the transformation of data and the extraction of descriptive outputs required for the MHQoL analyses. Therefore, the validation focused on ensuring that the toolbox reproduced the same results as the original statistical analyses. During development, one author implemented and documented all functions, after which a second author independently verified the outputs by reproducing the calculations and checking whether the toolbox produced results consistent with those obtained from the original analyses using example datasets. We have clarified this validation process in the manuscript.

8. *For the CFA model fit index, the RMSEA should be reported with 90%-CI.*

In line with recommended reporting practices for structural equation modelling, we have added the 90% confidence intervals for the RMSEA for all CFA models. These are now reported in Table 2.

9. *The manuscript relies heavily on Spearman's correlations; a discussion of potential common method bias (all self-report instruments) would be appropriate.*

To address this point, we have added a statement in the limitations section of the Discussion acknowledging that the MHQoL, MHQoL-VAS, and EQ-5D-5L are all self-reported measures and that this may contribute to shared method variance in the observed correlations.

10. *Some correlations between mental health instruments and MHQoL appear weaker than expected in Finland; the authors could explore cultural or translation-related factors.*

In the revised manuscript, we have expanded the Discussion section to address this point. Specifically, we now note that these differences may partly reflect the absence of a Finnish EQ-5D-5L value set, which required the use of the Danish value set as a proxy when calculating utility score, which has been further clarified in the discussion.

11. *Figures (e.g., regression plots) would benefit from confidence intervals to enhance interpretability.*

Both figures already include confidence intervals to illustrate the uncertainty around the estimated coefficients. We have reviewed the figures to ensure that these confidence intervals are clearly visible and interpretable for readers. Please point us to a specific figure if we have missed something.

Reviewer: 2

1. *In the abstract, the results should be more concisely reported in the results section and not repeat the methods. Measurement invariance testing does only make sense if you report the groups that were tested (in this case countries). Please report the groups in the abstract. Please also report the concrete (range of) Cronbach's alpha instead of the threshold in the results section.*

The Results section of the abstract has been revised to report the findings more concisely and avoid repetition of methodological details. We now explicitly report the countries included in the measurement invariance analysis (Finland, Spain and the United Kingdom) and provide the observed range of Cronbach's alpha values (0.741–0.806) instead of referring to a threshold.

2. *Authors report that validated versions in Finnish, Spanish and English were used. If so, they should also cite the validation studies of the related versions in the methods section accordingly.*

The language versions used in the study were official versions provided by the instrument developers. Specifically, the EQ-5D-5L translations were obtained from the EuroQol Group, and the MHQoL translations were provided by Erasmus University Rotterdam. As these are official validated versions provided by the developers, no separate validation publications are available to cite. We have clarified this in the Methods section.

3. *Please report the minimum and maximum age of the participants for each sample.*

The minimum and maximum ages for each country sample have been added in the population section of the manuscript (paragraph 2.2 population).

4. *The authors state "Gender did have an influence on the MHQoL score" and refer to Figure 2. Please report correctly to which gender the coefficient applies (or add the reference category) to make this information interpretable for the reader. This*

aspect should also be shortly discussed and compared to other studies on gender differences in mental quality of life.

During revision we recoded the gender variable to a binary variable (male vs female) for consistency in the regression models. In the previous version, gender had been included as a factor variable, and a small number of observations representing missing values had been coded as a separate category. Because gender was treated as a factor in the models, this additional category was not clearly visible in the previous figures, which led to a minor inconsistency in the gender calculations. After correcting this and re-estimating the models using only the male and female categories, gender was no longer significantly associated with MHQoL scores. This correction resulted in a very small change in the R^2 of model 2 (approximately 0.001), which has been updated in the revised figures and text.

5. *The authors describe limited generalizability due to mostly white-collar, female and higher educated participants. While that is certainly true, there may also be a selection bias. Descriptive statistics e.g. show low scores in anxiety, depression, etc. The authors should provide information about the sampling strategy of the original study. Since the original study contained an intervention, this could also have had impact on motivation to participate in the study. Even though the details may be described in the study protocol of the original study, a few more information about the sample (recruitment, response) should be provided in this study in the methods and limitation section (selection bias).*

To address this, we have expanded the Methods section to provide additional detail on the sampling and recruitment strategy of the EMPOWER trial. Specifically, we now clarify that organisations were recruited as clusters and that employees were invited to participate on a voluntary basis via their workplace.

Furthermore, we have added a statement in the limitations section acknowledging the potential for selection bias. In particular, we note that employees with a greater interest in mental health may have been more likely to participate.

6. *Baseline data from an intervention study were analyzed. Were follow-up data available to the authors to investigate test-retest reliability or responsiveness/sensitivity to change in mental quality of life? This would strengthen the results regarding psychometric validity in this study or could at least be mentioned for future investigations.*

We agree that assessing test–retest reliability would provide additional information on the temporal stability of the MHQoL. However, the present analyses were based on

baseline data only. Although the broader trial included follow-up measurements, these contained substantial missing data, which prevented reliable test–retest analyses. We have therefore clarified this limitation in the Discussion section, noting that the current validation is restricted to cross-sectional analyses and that future studies should further evaluate temporal stability and responsiveness.
